# Finite Element Analysis of Customized Acetabular Implant and Bone after Pelvic Tumour Resection throughout the Gait Cycle

**DOI:** 10.3390/ma14227066

**Published:** 2021-11-21

**Authors:** Leonid Maslov, Alexey Borovkov, Irina Maslova, Dmitriy Soloviev, Mikhail Zhmaylo, Fedor Tarasenko

**Affiliations:** 1 Institute for Advanced Manufacturing Technologies, Peter the Great St. Petersburg Polytechnic University, 29 Politekhnicheskaya, 195251 St. Petersburg, Russia; borovkov@compmechlab.com (A.B.); maslova.i@compmechlab.ru (I.M.); Solovyov.d@compmechlab.ru (D.S.); zhmaylo@compmechlab.com (M.Z.); tarasenko@compmechlab.ru (F.T.); 2 Department of Theoretical and Applied Mechanics, Ivanovo State Power Engineering University, 34 Rabfakovskaya, 153003 Ivanovo, Russia

**Keywords:** strength, computer simulation, finite element analysis, implant, pelvis, walking

## Abstract

The aim of this paper is to investigate and compare the stress distribution of a reconstructed pelvis under different screw forces in a typical walking pattern. Computer-aided design models of the pelvic bones and sacrum made based on computer tomography images and individually designed implants are the basis for creating finite element models, which are imported into ABAQUS software. The screws provide compression loading and bring the implant and pelvic bones together. The sacrum is fixed at the level of the L5 vertebrae. The variants of strength analyses are carried out with four different screw pretension forces. The loads equivalent to the hip joint reaction forces arising during moderate walking are applied to reference points based on the centres of the acetabulum. According to the results of the performed analyses, the optimal and critical values of screw forces are estimated for the current model. The highest stresses among all the models occurred in the screws and implant. As soon as the screw force increases up to the ultimate value, the bone tissue might be locally destroyed. The results prove that the developed implant design with optimal screw pretension forces should have good biomechanical characteristics.

## 1. Introduction

The human pelvis is a geometrically complex, biomechanical structure that carries the weight of the human body and stabilizes and protects inner organs. The pelvis can be damaged due to problems with the primary implant, infections, accidents, or bone tumours, which usually involve a large area of tissue removal and affect the patients’ lives. Due to the complex anatomical structure, the reconstruction of pelvic biomechanics after the loss of bone structure is still a challenge [1]. Various implants are used for different types of pelvis injuries, such as modular pelvis prostheses, saddle prostheses, pedestal cups, and custom-made pelvis prostheses. Among them, custom-made endoprostheses are matched with the patient’s bones, which in turn can reduce the risk of infection, dislocation, or failure of the implant [2]. Therefore, a custom prosthesis design is in demand when it is required to treat a complex bone fracture or replace a primary serial implant.

Previous research [3] presented pelvis reconstruction by applying a fibula and a variation of the methods of internal fixation of the implant. In this study, a vertical load of 500 N was applied to the L3 lumbar vertebrae, and the pelvis was considered to be in a bipedal standing position. As a result, the stress concentration in the fibula implant was extremely high, but this effect was minimized by internal fixation, which partially transferred the stresses from the fibula to the screw system. Additionally, a high stress concentration was detected in the implant. Among the four methods of fixation, the best method was a double rod system with an L5-S1 pedicle and iliac screws, which provided the lowest stress concentration and the lowest displacement of the pelvis.

A previous study [4] describes a modular endoprosthesis for the damaged half of the pelvis. In the course of this research, a comparative analysis of the stress distribution between the healthy and reconstructed pelvis was carried out in three static positions: sitting, standing, and standing on the foot of the injured side. The loads and boundary conditions were similar to those described in the study above [3]. In the healthy pelvis, the stress distribution was concentrated in the upper region of the acetabulum, arcuate line, sacroiliac joint, sacral midline, and, in particular, the upper region of the greater sciatic notch. In the reconstructed pelvis, the stress distribution was concentrated on the proximal area of the pubic plate, the top of the acetabulum, the connection between the CS fixator and acetabular cup, and the fixation between the prosthesis and sacroiliac joint. The stress distribution in the reconstructed pelvis was similar to the stress distribution in the healthy pelvis in the three different static positions.

Generally, the clinical efficacy and biomechanical features of the implants used for pelvic injuries should be evaluated through biomechanical experiments in vitro. However, irregular geometry and material heterogeneity of the pelvis often make mechanical experiments challenging [5].

In modern orthopaedic biomechanics, a computational approach was developed for analysing the stress and strain distributions of a hip joint endoprosthesis [6]. The study under consideration is based on the finite element (FE) method to investigate stresses on the bones and implant.

The FE method has proven to be a powerful tool in reducing the cost and time in many biomechanical studies and has become an important tool for understanding overall biomechanical behaviour. Nevertheless, many factors, such as material properties, anatomical geometry, the integrity of the human structure, and boundary conditions, could influence the accuracy of FE results [7].

Thus, the FE method is becoming increasingly popular in pelvis biomechanics research and plays a critical role in failure analysis and revision prosthesis design [2]. Although some FE analyses of custom-made prostheses have been carried out, studies of the influence of the prestress of the screws on the biomechanical performance of a reconstructed pelvis for walking patterns are rarely reported [2].

The aim of this study was to investigate the stress distribution of the pelvis reconstructed by individual endoprostheses with different screw forces and then identify which force value is optimal for tightening the implant and the bone. After that, the stress distribution in the “bone–endoprosthesis” system was obtained for typical walking loads and chosen screw forces.

## 2. Materials and Methods

### 2.1. Finite Element Models

**Figure 1:**
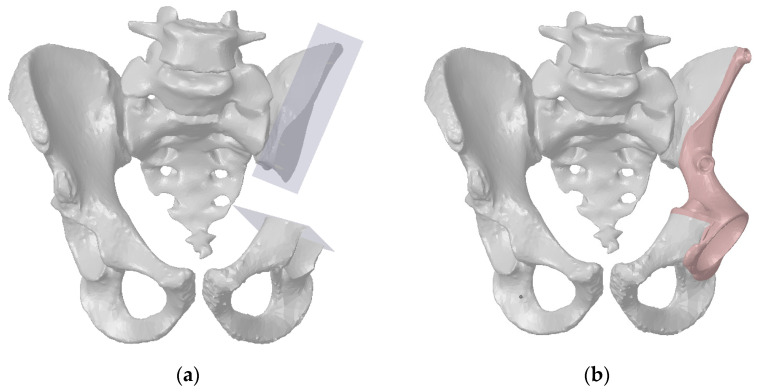
A virtual resection of the pelvic bones for pelvic tumour surgery: (**a**) resection planes; (**b**) the pelvis reconstructed with the individual endoprosthesis.

Three-dimensional reconstruction was performed for the case of a young patient whose weight was 50 kg. He underwent treatment at the Federal State Budgetary Institution National Medical Research Center of Oncology named after N. N. Blokhin of the Ministry of Health of the Russian Federation (N.N. Blokhin NMRCO). Three-dimensional models of the patient’s pelvis were obtained using next-generation, multi-slice computed tomography with high resolution and innovative software [8]. These 3D models were provided by N. N. Blokhin NMRCO (Figure 1). The CAD models consisted of several faceted surface bodies.

**Figure 2:**
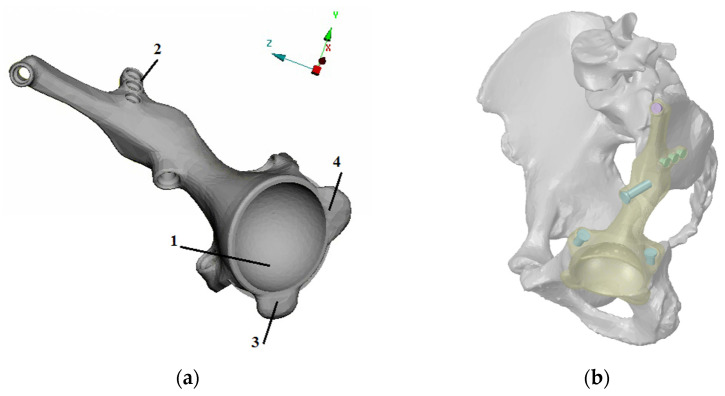
Developed design of the individual endoprosthesis considered in this paper: (**a**) design and main components of the implant: the cup (1), the bearing flange on the iliac bone (2), the bearing flange on the pubic bone (3), and the bearing flange on the ischiatic bone (4). (**b**) Implant position and fixation by seven screws.

The abovementioned CAD models were the basis for developing a computer model of the individual endoprosthesis. The design of the customized endoprosthesis is shown in Figure 2. The main parts of the implant are the cup (1), the bearing flange on the iliac bone (2), the bearing flange on the pubic bone (3), and the bearing flange on the ischiatic bone (4).

**Figure 3:**
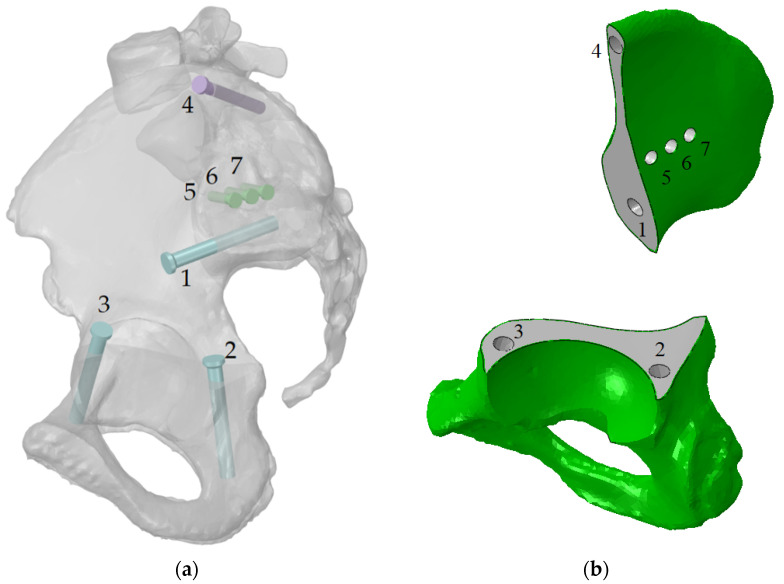
Implant fixation: (**a**) the screws numbered from 1 to 7 and their positions (the implant is hidden); (**b**) the holes numbered from 1 to 7 in the bone parts, the same numeration as this for the screws.

The personalised implant fastened to the damaged parts of the pelvic bones with seven screws (Figure 3). The drilled holes, which are also shown in the figure, have the same numbering as the screws. Screws with numbers from 1 to 3 have a length of 55 mm and diameter of 6.5 mm, and screws from 5 to 7 are 15 mm long and have a diameter of 4.5 mm. Screw 4 has a length of 45 mm and diameter of 5.5 mm.

The development of models for numerical analysis is a relatively complicated process. Medical researchers are always concerned about verifying analytical models because incorrect assumptions in the FE model might lead to an incorrect stress distribution [9,10,11,12,13]. For this reason, a great amount of time was devoted to the development of the FE model, in particular the choice of the FE types and the mesh grid density.

**Figure 4:**
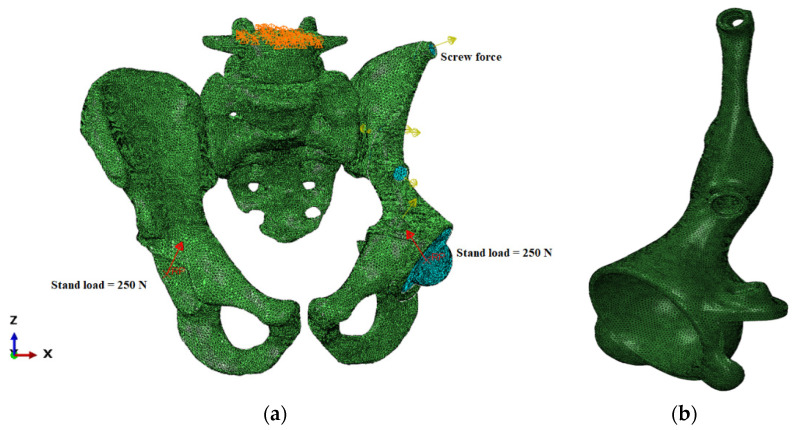
Finite element model of the “bone–endoprosthesis” system: (**a**) finite element mesh on the pelvis; (**b**) finite element mesh on the implant.

Additionally, it was necessary to consider the performance of the computer while defining the parameters of the finite element models. The current study was performed using an ordinary workstation, and the mesh grid parameters were chosen in such a way that the analysis model could be successfully run on the machine. The total number of finite elements in the assembly amounted to 1,203,061 elements of the mesh prepared in ABAQUS/CAE software (Dassault Systems Simulia Corp., Johnston, RI, USA) (Figure 4).

The finite element size for the pelvic bones and implant ranged from 0.5 mm to 2 mm. It complied with the conclusion of previous research [2] studying the dependence of the optimal mesh size and obtaining reliable results. It should be mentioned that the cortical layer was modelled as a solid body with a constant thickness by offsetting the outer surface inward by 0.5 mm (Figure 3b). The characteristics of the FE model are shown in Table 1.

The quality of FE models is verified by three criteria: aspect ratio, skewness, and warping. The verification showed that the general quality of the mesh was relatively high, but some elements did not satisfy the quality criteria, as indicated in Table 1. Note that according to the aspect ratio and warping criteria, no poor-quality elements were found. The poor-quality elements appeared due to the strict skewness criteria. However, at current skewness settings, the maximum error is no more than 3.5% of the total quantity of the finite elements. So, the amount of such elements was quite low, and this fact could be neglected without loss of accuracy.

### 2.2. Material Properties

Particular attention should be given to the physical and mechanical properties of the bone tissue. The pelvic bone consists mainly of low-density spongy tissue and a thin and dense cortical layer. Most of the load is transferred through the cortical layer, and the spongy tissue works as a support material, preventing the cortical layer from collapsing. Due to age and other reasons that may cause degradation of bone tissue, the mechanical properties can change [14,15,16,17]. Bone tissue is anisotropic [12,14,18,19], and the distribution of Young’s modulus depends linearly on the density. However, the variation in Young’s modulus both for the compact and spongy tissue is negligible for the pelvic bone, as proven previously [9,14]. Many papers devoted to the pelvis finite element study considered the pelvic bone as isotropic and linear elastic [1,2,3,4,5,6,7,8]. After analysing the data from these sources, the mechanical properties are summarised in Table 2.

It is important to mention that the yield strength reported previously [12,13,14] for spongy bone seems excessively high. From a clinical point of view [20], the spongy bone has a yield strength of approximately 1%, so some additional evaluation is required to confirm that the spongy bone could be loaded up to 2–3% of strain without any plastic deformation, damage, or fracture. Therefore, a value range of 1.4–2.1 MPa was used in this study as the ultimate strength of the spongy bone. Data reported previously [20] were confirmed by the authors’ research on spongy tissues extracted from the femoral head after surgery [15].

The next important point is to study the material characteristics of the other parts of the assembly. The main advantages of titanium alloy Ti-6Al-4V are good corrosion resistance under all conditions and excellent biocompatibility.

The screws and prosthetic head should be made of normal solid titanium alloy Ti-6Al-4V [21], and the considered implant is manufactured by 3D printing [22]. The mechanical properties of 3D printed and solid titanium are slightly different, particularly those characterizing the fatigue behaviour [23].

Finally, the acetabular liner is made of ultrahigh-molecular-weight polyethylene [24]. This polyethylene, reinforced with chemical cross-links, is distinguished by its strength, low friction coefficient, and high biocompatibility. These properties allow using it for artificial joints.

### 2.3. Loads and Boundary Conditions

Boundary conditions and contact interactions were defined in ABAQUS/CAE software (Dassault Systems Simulia Corp., Johnston, RI, USA).

The solution of the task in ABAQUS included two steps: screw tightening and standing. The region of the contact interaction and boundary conditions were unchanged between the steps. The model had contact interactions with friction in the following pairs: the implant and pelvic bone and the acetabular liner and the artificial femoral head. The coefficient of friction was equal to 0.2 for the titanium–bone pair and 0.15 for the polyethylene–titanium pair. The screw heads and threads are bonded with implant and bone correspondingly, so these contact pairs are considered as linear contacts without friction and separation.

The first step was to bring together the implant and the pelvic bone with the screws. Therefore, a special compressive screw force was applied to each screw. The calculations were conducted with four values of screw forces: 100, 500, 1000, and 1500 N. The upper surface of the sacrum is fully constrained.

**Figure 5:**
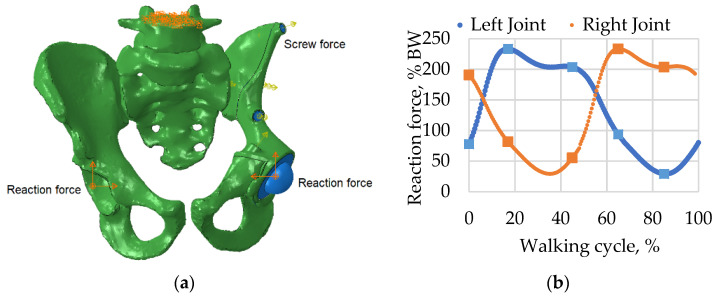
Boundary conditions and applied forces: (**a**) model with boundary conditions and loads including screw pretension and reaction forces applied to the centres of the left and right joints; (**b**) reaction force curves for walking simulation measured as a percentage of body weight (BW).

The second step simulated the walking cycle as a slow, quasistatic process. The reaction forces corresponding to the patient’s weight were applied to the reference points in the centres of the acetabular cups (Figure 5). The reaction force was obtained from the HIP98 software available from the OrthoLoad open resource (https://orthoload.com/test-loads/data-collection-hip98/, accessed on 1 October 2021). The software generates the biomechanical forces based on the special database, which was developed within study [25] using special instrumented implants.

At the beginning of the second loading step, where the walking process was simulated, the length of the screws was fixed, which occurs at the end of the pretension phase. A detailed strength analysis was further performed for screw force values of 500 N and 1000 N. The general kinematic boundary conditions remained unchanged from the previous step of the considered computer simulation.

### 2.4. Model Validation

The FE method requires strict validation of the model because an inaccurate model may lead to incorrect and unreliable results [9,10,11]. Comprehensive experimental validation of the FE model of the reconstructed pelvis is not carried out before the surgery because a definite forecast of the bone stress state is required before performing surgery.

However, the evaluation of the general adequacy of the model was carried out in accordance with several parameters. First, the FE mesh was assessed based on several criteria (aspect ratio, skewness, warping), and mesh quality was evaluated based on element size, type, and shape. Second, the physical and mechanical characteristics of materials ensuring model accuracy were obtained from reliable sources [9,12,14,16,18,19].

Kinematic boundary conditions for the model and contact regions were applied and approved by the medical studies described in other articles [11,26,27]. The loading scheme, which was used in current research and based on the loading of the structure with the hip joint force acting as a reaction force, was the same as used previously [28]. It allowed us to describe the stress-strain state in a more accurate way.

The preliminary frequency analysis proved that the reconstructed pelvis assembly was joint and adjusted correctly, and all the connections were set up properly.

**Figure 6:**
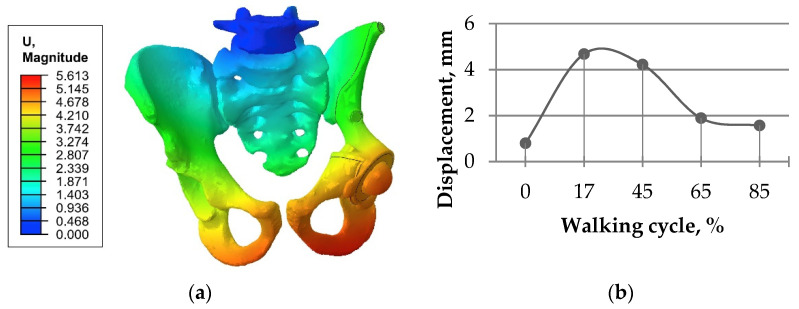
Total displacement in the “bone–endoprosthesis” system for the walking condition and a screw load of 1000 N: (**a**) typical displacement distribution (mm); (**b**) displacement values for the full gait cycle at the spherical center of the left femoral head.

Another validation based on the displacement distribution (Figure 6) showed that the values obtained in the current study were within range close to the ranges described in papers [1,10,29]. The results of the analysis of the stress and strain fields were also close to previous results [1,2,10], proving the principal qualitative and quantitative conformance.

## 3. Results

In this study, the problem of assessing the screw force effect on overall stress distribution is solved for the four values of pretension load equal to 100, 500, 1000, and 1500 N, and reliable results were obtained. Based on these results, a detailed analysis of the structural strength in the case of slow walking is presented below with emphasis for the screw force values of 500 and 1000 N.

### 3.1. Results for the Stage of Tightening the Screw Simulation

In the beginning of the study, the initial step of screw pretension was analysed, and the optimal value for the screw force was chosen. Since there was no specific value for the pretension force, it was decided to investigate this point in more detail. For the final assembly, the calculations were carried out with several values of the pretension force from 100 N up to 1500 N, and the force values were considered incrementally.

**Figure 7:**
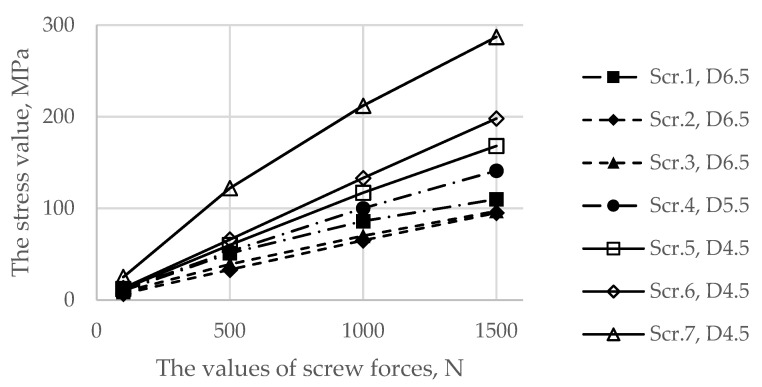
Dependence of the maximum von Mises stress in the screws as a function of the applied preload values.

**Figure 8:**
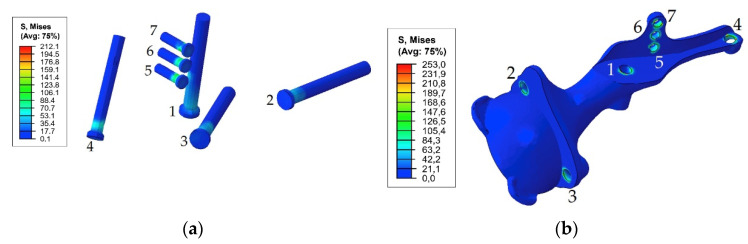
Equivalent von Mises stress (MPa) distribution caused by a pretension load of 1000 N: (**a**) in the screw system; (**b**) in the implant.

Figure 7 demonstrates the dependence between the maximum stress in each of the bolts and the applied tension force. The typical stress distribution in the screws and implant itself caused by the screw preload force of 1000 N is shown in Figure 8.

**Figure 9:**
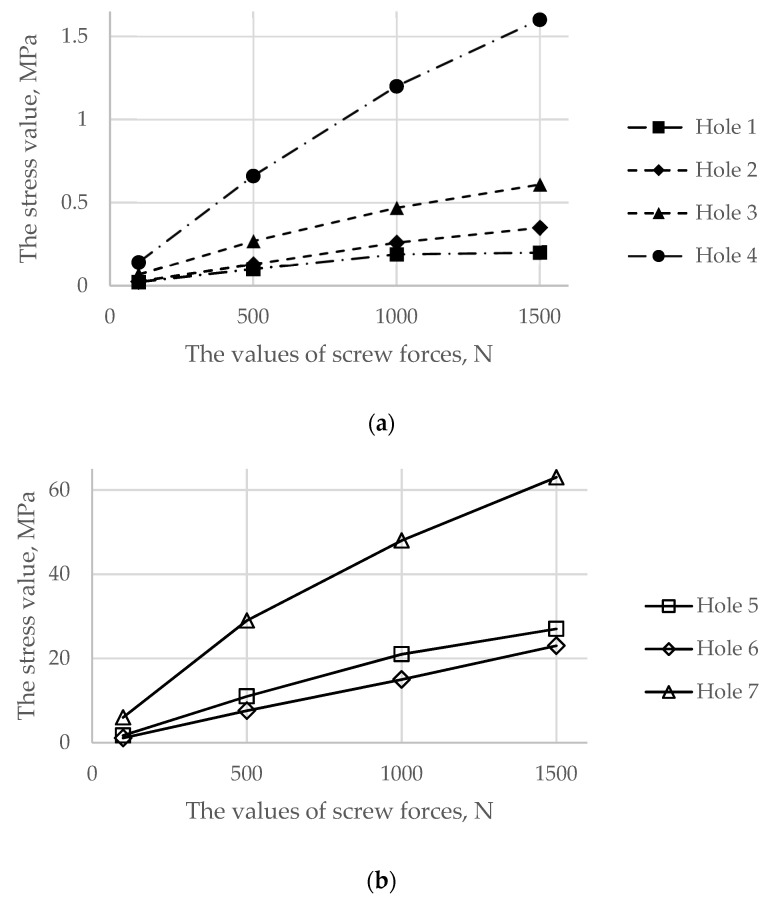
Comparative graphs of the maximum von Mises stresses occurring in the reconstructed pelvis bone on the hole boundaries: (**a**) the spongy tissue; (**b**) the cortical tissue.

Particular attention should be given to the stresses in the pelvic bones because the destruction of the bone might be initiated due to the high value of the screw force. Figure 9 represents comparative graphs of the maximum stress values arising in the spongy (a) and cortical (b) layers in the first row of the finite elements of holes 1, 2, and 3 in the spongy tissue and holes 5, 6, and 7 in the cortical tissue.

**Figure 10:**
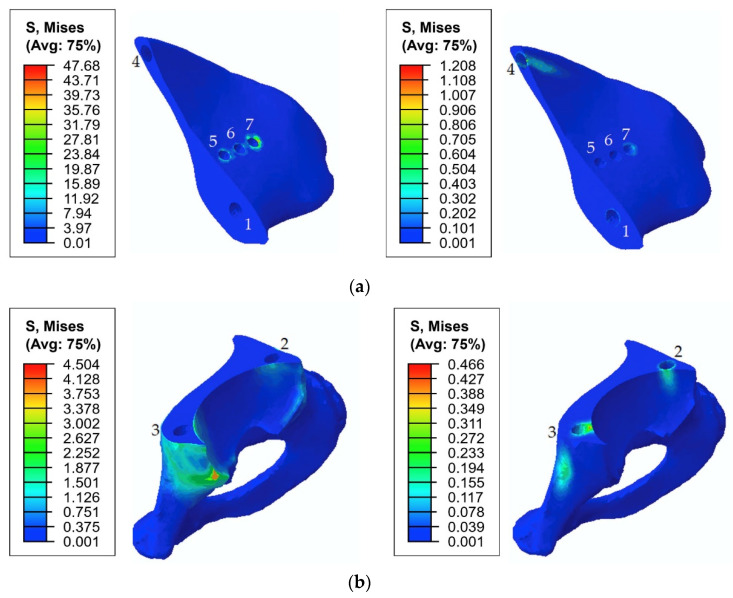
Equivalent von Mises stress (MPa) distribution in the bone tissue caused by a pretension load of 1000 N: (**a**) the upper part of the resected pelvic bone; (**b**) the lower part of the resected pelvic bone. The images on the left show the stresses in the cortical tissue, and the images on the right show the stresses in the spongy tissue.

Figure 10 shows the typical stress distribution around the holes in the resected pelvic bone after surgery.

The comparison was carried out separately for the spongy and cortical layers because screws with numbers 1, 2, 3, and 4 interacted only with the spongy layer. However, screws with numbers 5, 6, and 7 crossed the cortical layer. The screw with number 4 entered the spongy tissue but was located very close to the cortical layer.

As shown in Figure 8 and Figure 9, the maximum von Mises stress values that occurred in screw 7 and holes 4 and 7 in the case of a pretension force of 1500 N were close to the ultimate stresses according to Table 2. A significant change in the stresses took place near the holes, as shown in Figure 10, while the remaining volume of the bone was almost free from the stresses.

The analysis proved that a pretension force of close to or higher than 1500 N may lead to local bone fracture, and further analysis of the walking simulation made no sense. The optimal screw force was expected to be between 500 N and 1000 N, since it did not cause any bone destruction but provided sufficient contact between the bone and implant. Therefore, the following strength analysis of the “bone–endoprosthesis” system was carried out for a walking condition assuming screw forces equal to 500 N and 1000 N.

### 3.2. Results of the Walking Cycle Simulation

The detailed walking FE simulation was carried out for the prepared model with screw pretension values equal to 500 N and 1000 N as follows from the previous section.

**Figure 11:**
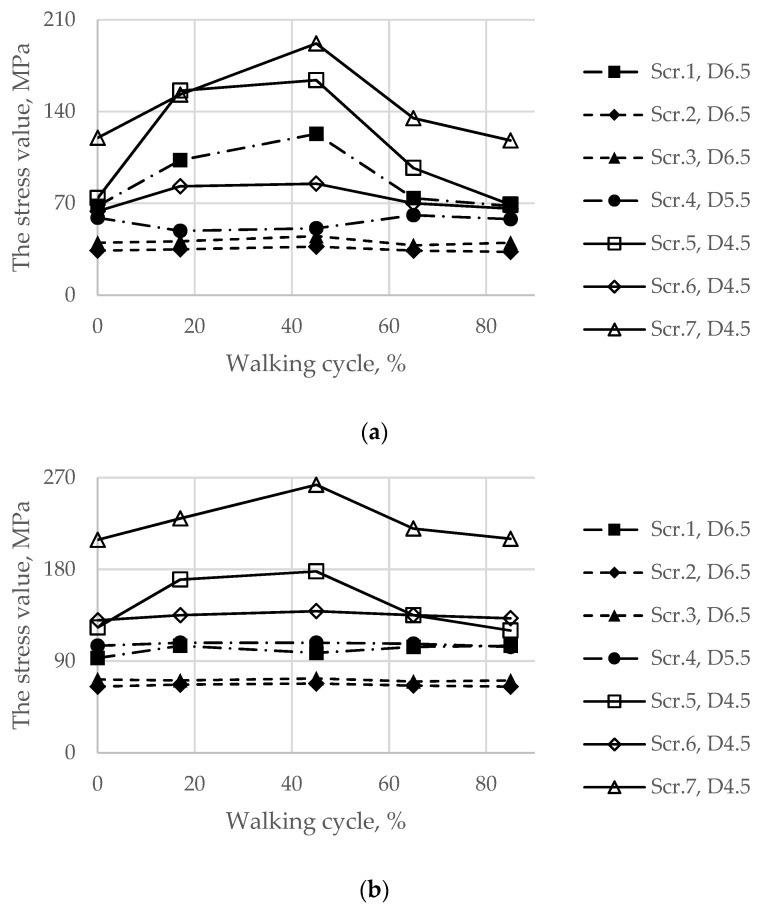
Maximum von Mises stress in each of the screws with pretension in the walking cycle phases: (**a**) pretension force equal to 500 N; (**b**) pretension force equal to 1000 N.

First, Figure 11 shows the von Mises stresses in the screws preloaded with forces of 500 and 1000 N, while the hip joint reaction force altered according to the graph in Figure 5 that simulates the gait cycle.

In addition, for the initial stage of screw pretension, the stress state in the titanium parts of the endoprosthesis in the walking condition was also considered. Particular attention was given to the load case corresponding to 17% of the gait cycle because this phase is the phase of the maximum reaction force applied to the centre of the left joint, where the implant was placed. However, obviously, extremely high stresses in the screws occurred at 45% of the gait cycle (Figure 11).

**Figure 12:**
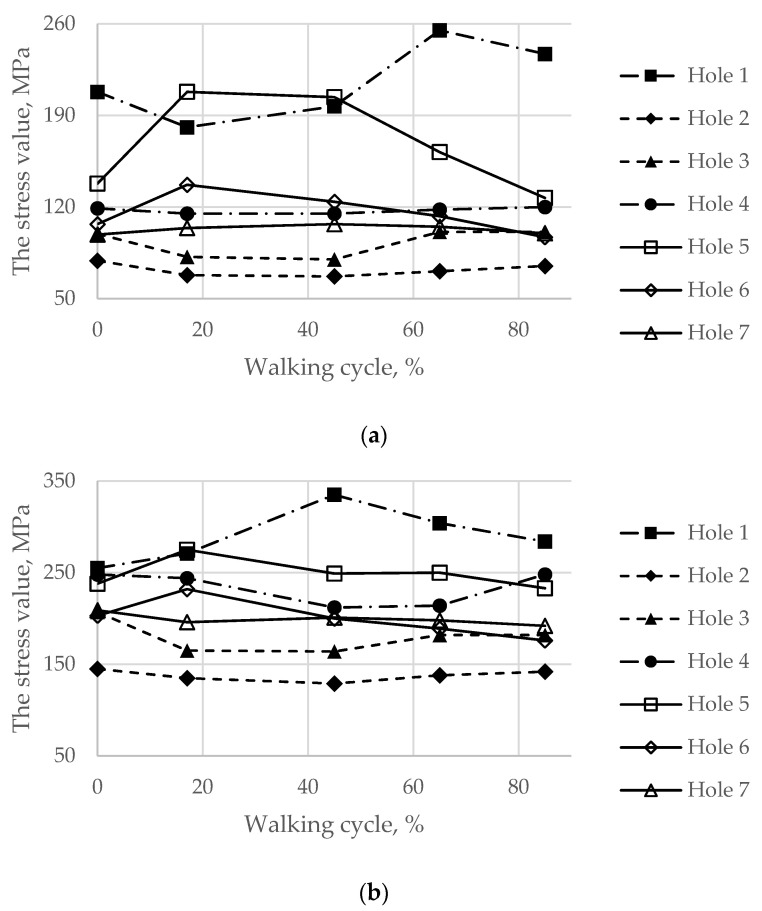
Maximum von Mises stress in the implant on the boundaries of the holes in the walking cycle phases: (**a**) pretension force equal to 500 N; (**b**) pretension force equal to 1000 N.

Similarly, the peak von Mises stresses for the considered walking phases were expected on the hole edges (Figure 12). The highest stresses in the implant took place at 45% of the gait cycle in the case of a 1000 N preload but at 17% and 65% for a 500 N preload.

**Figure 13:**
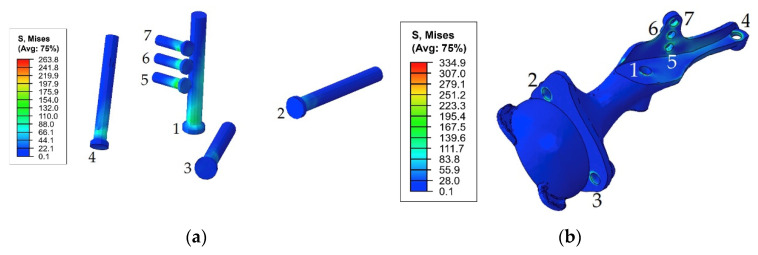
Equivalent von Mises stress (MPa) distribution in the endoprosthesis titanium parts caused by the pretension load of 1000 N and joint reaction forces at 45% of the gait cycle: (**a**) screw system; (**b**) implant body.

The general view of the typical stress distribution in the endoprosthesis is shown in Figure 13 for a preload of 1000 N and gait cycle phase equal to 45%.

**Figure 14:**
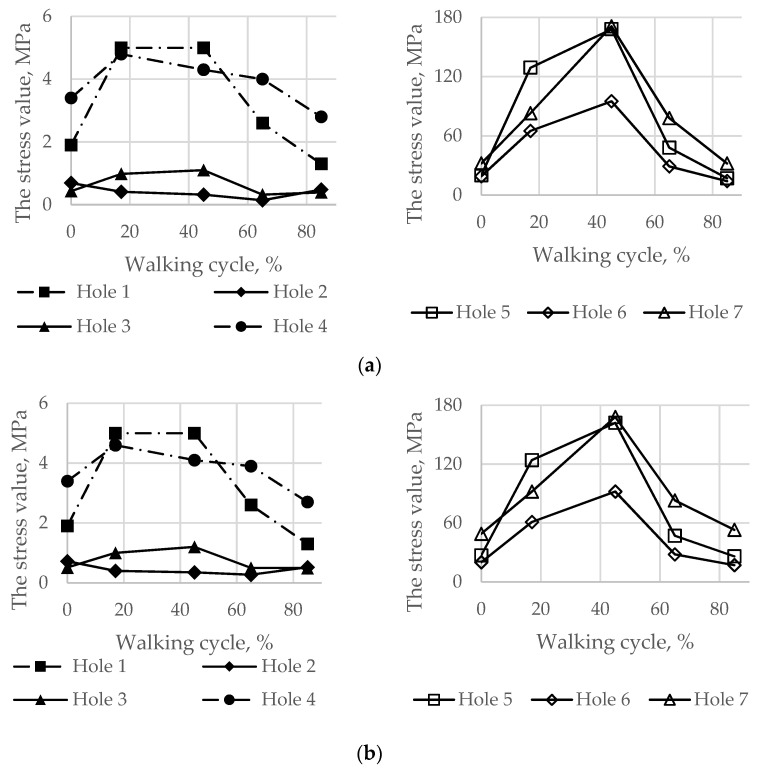
Maximum von Mises stress in the bone tissue on the boundaries of the holes шт in the walking cycle phases: (**a**) the pretension force equal to 500 N; (**b**) the pretension force equal to 1000 N.

Finally, the maximum von Mises stresses evaluated on the boundaries of the holes for the screws at every load point in the walking cycle are shown in Figure 14.

**Figure 15:**
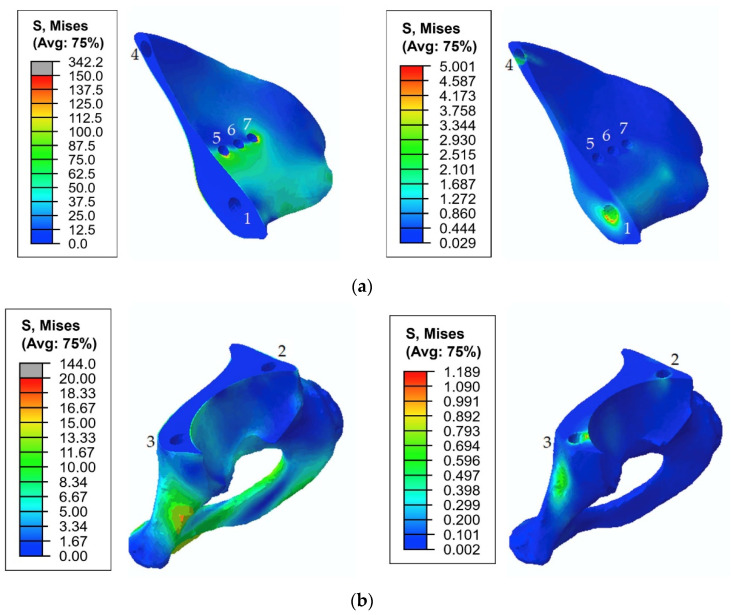
Equivalent von Mises stress (MPa) distribution in the bone tissue caused by the pretension load of 1000 N and joint reaction forces at 45% of the gait cycle: (**a**) the upper part of the resected pelvic bone; (**b**) the lower part of the resected pelvic bone. The images on the left show stresses in the cortical tissue, and the images on the right show stresses in the spongy tissue.

The total stress distribution that occurred in the pelvic bones for the typical gait phase was 45%, and the screw force of 1000 N is presented in Figure 15.

## 4. Discussion

For a more convenient assessment and comparison of the obtained results, a summary of the maximum von Mises stresses (MPa) occurring in the endoprosthesis parts and bone tissue are presented in Table 3 for the two considered values for the screw pretension loads: 500 and 1000 N.

The first step in the assessment of the long-term strength and reliability of the biomechanical “bone–endoprosthesis” system is to evaluate the mechanical strength of the implant and its fixation system. According to Figure 7, the stresses occurring in the screws vary mostly linearly depending on the applied pretension force. This finding allows us to obtain the required force in a relatively simple way. The high stresses are distributed quite locally. The highest stress level takes place in the areas of contact between the screws and the screw holes in the implant (Figure 8) and the bone tissue (Figure 10).

However, it should be mentioned that the real stresses in the screws differ significantly from the tensile stress in the metal rod loaded with a similar tensile force, which is calculated as the ratio between the force and the cross-sectional area. For example, for screws 5, 6, and 7 with a diameter of 4.5 mm loaded with a longitudinal force of 1000 N, the sectional stresses are equal to 63 MPa, whereas the total equivalent stresses considering the contact interaction reach values from 117 to 212 MPa. This fact emphasizes the importance of considering the contact interaction of the bodies of the “bone–endoprosthesis” system both for the first stage of screw pretension and for the subsequent walking step.

According to Table 3, the screws and implant at pretension values of 500 N and 1000 N have a safety factor of more than 4.0 based on data in the literature [3] (Table 2). The Mises equivalent contact stresses on the edges of the screw holes in the implant are close to the total stresses in the corresponding screws. However, the stresses in the implant are slightly lower. The difference approximately equals the pretension stresses in the screws.

Analysis of the static strength of the screws allows us to assume that there should be no destruction of the titanium components of the system when the patient is walking. However, this assumption becomes debatable in further analysis of the structural strength under periodic loads occurring in the hip joint during normal human activity.

In the case of walking, the development of fatigue damage is highly possible on the edges of the implant holes for both considered options for screw tightening (Figure 12 and Figure 13). The maximum equivalent stresses exceed the lower bound of the fatigue limit for 3D printed Ti-6Al-4V, which is 200 MPa according to the obtained material data (Table 2). At a pretension force of 500 N, the stresses exceed the limit only in the region of hole 1, whereas at a force of 1000 N, the limit is exceeded for all holes except hole 2. However, most likely, the risk of fracture due to fatigue effects may decrease with successful osseointegration and should not have any significant effect thereafter. Regardless, the use of high-quality titanium powders and the application of advanced manufacturing technologies for producing implants [22] should have a high priority in the planning stage for such surgeries.

In the fixation system, screws 1 and 4–7, which fix the implant to the upper part of the pelvic bone, are mostly affected by the periodic loads that occur during walking. In screws 2 and 3, the stresses do not exceed 80 MPa since these screws do not carry any significant external load. However, these screws cannot be excluded from the system because they connect the implant and the lower part of the pelvic bone, reconstructing the pelvic ring. In all screws, the maximum stresses did not reach the lower bound of the fatigue limit for Ti-6Al-4V according to the obtained material data (Table 2). The highest stress value equals 263 MPa and occurs in screw 7.

The analysis of the stresses in the bone tissue of the analysed biomechanical system requires special attention. As mentioned above, at the stage of screw tightening, the equivalent von Mises stresses on the edges of the screw holes in the cortical and spongy tissues (Figure 9) for tightening forces of 1000 N and above approach the lower bound of the strength limit of the corresponding tissue (Table 2) but do not reach the critical values. The cortical tissue of the upper pelvic bone has a safety margin against the strength limit at the preloading stage for all the considered values of the pretension forces. The spongy tissue also has a significant safety margin in the case of the pretension of 500 N. In the case of 1000 N, the stresses are close to the limit but do not exceed it. The remaining regions of the bone remain mostly unstressed, which is quite reasonable due to the chosen detailed setup of the problem.

In the cortical layer of the lower part of the pelvis, maximum stresses are situated in the pubic joint. These stresses are caused by the rigid connection of the left and right pelvic bones in this area. In other areas, at all stages of the simulation, stresses do not exceed 20 MPa, and there is a safety margin of at least 4.0. A similar situation occurs in the area of the rigid connection between the upper part of the pelvic bone and the sacrum. The increased stresses in these areas might be neglected because they are caused by artificial rigid constraints and occur due to the absence of the soft cartilage layer in the model. Furthermore, these areas are not subjected to surgical intervention, and their physiological condition remains unchanged compared with the healthy biomechanical system of the pelvis, where there are no high mechanical stresses that exceed the strength limits during normal human activities [30].

During the walking phase, areas with extremely high stresses, which exceed the allowed limits, appear both in the cortical and spongy tissues in the upper part of the resected pelvic bone and for both pretension forces considered (Figure 14 and Figure 15). According to Table 3, the most dangerous regions where local destruction might be expected are the edges of screw holes 1 and 4 (spongy layer, equivalent stress of 4–5 MPa) and holes 5 and 7 (cortical layer, equivalent stress of 160–170 MPa). The limits are significantly exceeded in the walking cycle phases of 17% and 45%. The stress near hole 6 equals 95 MPa and exceeds the lower limit of the allowable stress range (80 MPa). This fact confirms the high risk of bone damage.

The stress values obtained in the “bone–endoprosthesis” system during the walking simulation are slightly higher than the stress values reported previously [28], where a similar approach was used. The reported peak stresses in the implant were approximately 105 MPa during the walking phase, and the stresses in the screws and pelvic bones were approximately 50 MPa under the same loading condition. Therefore, the authors [28] expected that the fatigue limit could be reached only in the case of more severe loading scenarios, such as stair climbing. The difference in the results can be explained by the difference in the implant design and the differences in the approaches of finite element modelling of the behaviour of the bone tissue.

Thus, according to the performed analysis, slight destruction of the bone might be expected in local areas near the screw holes in cases of walking. This destruction can affect the stability of the implant fixation to the upper bone. However, bone tissue is capable of regeneration when it is loaded with an external mechanical field, in particular, with periodic loading of a sufficient level. Therefore, in the case of a moderate dynamic compressive load, the process of regeneration could be initiated in the area that might be initially damaged [31].

The current study particularly focuses on the screw tightening process and on the analysis of the values of the forces applied to the screws. This issue is usually neglected in studies, and the number of related publications is very limited [2,3,4]. Nevertheless, this point is quite important for proper patient surgery planning. The degree of fixation between the implant and the bones directly depends on the value of the screw pretension force. Additionally, fixation affects the reliability of the reconstructed pelvis in terms of cyclic loading and fatigue effects. If the screws are tightened loosely, then the process of bone regeneration might take much more time. If the screws are tightened excessively, local destruction will occur in the bones near the holes. This fact can lead to more serious consequences over time. Considering previous results [2], it should be pointed out that the screw pretension force equals 3000 N. However, according to the current study, increasing the screw tightening force from 1000 to 1500 N may lead to local bone destruction. Comparison of the obtained results with the charts provided [2] shows the compliance of stresses for tightening forces up to 500 N. The results from previous research [2] also confirm the linear growth of stresses in the screws.

The shape of the endoprosthesis is customized, which causes additional problems for the stress-strain state analysis of the structure. It should be mentioned that the methods of designing individual implants differ in complexity from the methods of developing serial endoprostheses [1]. Additionally, the design of the individual endoprosthesis can change during the modelling process. However, the design of serial implants is usually as efficient as possible, as opposed to individual implants, which are designed and produced only once for a specific patient. Using the finite element method, it is possible to predict the areas of stress concentrations and to choose the optimal number and parameters of the screws. In the current study, there were several screws that did not carry much load. This finding indicates that they may be removed from the structure. The authors presume that if the stress concentration does not occur in the screws, such action should not affect the general performance and reliability of the structure. A previous study [32] confirms this statement, saying that such screws accumulate excess stresses and should be removed.

When the current stress-strain state results are compared to results from similar studies [1,2,10], it should be considered that the forces applied to the structure are not universal and vary depending on the patient. For the stress-strain state comparison [2], the stress concentration arises around holes, and the stress values match the results of current research. The same trend takes place in other studies [11], especially for cup-shaped structures.

## 5. Conclusions

The stress distribution for a pelvis reconstructed by an individual endoprosthesis with four different screw forces was analysed. The obtained optimal screw pretension force for tightening the implant to the bone was from 500 N to 1000 N for this specific model.

Screw tightening with a force less than 500 N seemed to be insufficient for firm fixation of the implant. At the same time, the results show that a tightening force exceeding 1000 N may result in a local bone fracture. Therefore, the optimal and critical screw forces are determined, and the stress states are calculated for the walking condition. The peak stresses occur near the holes in the bones, implant, and screws. Screw tightening with a force of 500–1000 N should be optimal because the stress state of the bones did not exceed the limits globally. This value for screw force provides reliable fixation of the implant to the bones.

When conducting the subsequent surgery, it is strongly recommended to monitor the value of the actual screw pretension force. In this case, the endoprosthesis will be reliable and durable. To prevent the undesirable development of degenerative effects during the patient’s recovery process after osteosynthesis surgery, the rehabilitation plan should be adjusted to reduce the loads on the reconstructed bone by providing additional support when the person is walking.

As a result of the arthroplasty described in current research, the patient has fully recovered with no limitations in motion or activities [8]. This fact confirms the relevance of the performed studies and the significance of further development of computer modelling methods and approaches for solving the problems of personalized orthopaedics. The technology of implant development using computer modelling, finite element analysis, and 3D printing makes it possible to create anatomical prostheses with sufficient safety margins, anatomical designs, and reliable fixation methods.

## Figures and Tables

**Table 1:** Characteristics of the finite element model.

Part	Number of Finite Elements	Finite Element Type	FEM Verification		
			Aspect	Skewness	Warping
Top of the damaged half of the pelvic bone	77,392	Four-node linear solid tetrahedral C3D4 type	No violations	2892 elements off	No violations
Bottom of the damaged half of the pelvic bone	122,131			3031 elements off	
Healthy pelvic bone	265,775			8807 elements off	
Sacrum	44,746			2009 elements off	
Implant	329,828			12,615 elements off	

**Table 2:** Material properties used in the present study.

Material	Young’s Modulus, GPa	Poisson’s Ratio	Ultimate Stress, MPa	
			Yield	Fatigue
Cortical tissue [9,18]	17	0.3	80–150 [18]	the same as the yield stress
Spongy tissue [14,20]	0.07	0.2	1.4–2.1 [20]	
Normal Ti-6Al-4V [21]	113.8	0.34	950	310–610 [21]
3D printed Ti-6Al-4V [22,23]	123.4	0.26	910	200–500 [23]
Polyethylene [24]	1	0.35	26	-

**Table 3:** Summary of the maximum von Mises stresses occurring in the endoprosthesis parts and bone tissue for screw pretension loads equal to 500 N and 1000 N.

Assembly Components	Maximum Von Mises Stresses (MPa) and Their Location			
	Pretension Force of 500 N		Pretension Force of 1000 N	
	Pretension Stage	Walking Cycle (45% Phase)	Pretension Stage	Walking Cycle (45% Phase)
Screw system	122 MPa, screw 7	192 MPa, screw 7	212 MPa, screw 7	263 MPa, screw 7
Implant	151 MPa, hole 1	197 MPa, hole 1 *255* MPa, *hole 1* (*65*% *phase*)	253 MPa, hole 4	335 MPa, hole 1
Pelvic cortical tissue, top part of resected bone	29 MPa, hole 7	171 MPa, hole 7 168 MPa, hole 5	48 MPa, hole 7	168 MPa, hole 7 162 MPa, hole 5
Pelvic spongy tissue, top part of resected bone	0.68 MPa, hole 4	4.98 MPa, hole 1 4.3 MPa, hole 4	1.25 MPa, hole 4	5.0 MPa, hole 1 4.1 MPa, hole 4
Pelvic cortical tissue, bottom part of resected bone	2.7 MPa	19 MPa	4.5 MPa	19 MPa
Pelvic spongy tissue, bottom part of resected bone	0.28 MPa, hole 3	1.1 MPa, hole 3	0.47 MPa, hole 3	1.2 MPa, hole 3

## Data Availability

The data presented in this study are available on request from the corresponding author.
